# The Nursing Diagnosis of risk for pressure ulcer: content validation**^1^**

**DOI:** 10.1590/1518-8345.0782.2693

**Published:** 2016-06-14

**Authors:** Cássia Teixeira dos Santos, Miriam de Abreu Almeida, Amália de Fátima Lucena

**Affiliations:** 2 RN, Hospital de Clínicas de Porto Alegre, Porto Alegre, RS, Brazil. Profeasor, Centro Universitário Metodista de Porto Alegre, Porto Alegre, RS, Brazil.; 3 PhD, Associate Professor, Escola de Enfermagem, Universidade Federal do Rio Grande do Sul, Porto Alegre, RS, Brazil.

**Keywords:** Pressure Ulcer, Nursing Diagnosis, Nursing Process, Risk Factors, Nursing

## Abstract

**Objective:**

to validate the content of the new nursing diagnosis, termed risk for pressure ulcer.

**Method:**

the content validation with a sample made up of 24 nurses who were specialists in skin care from six different hospitals in the South and Southeast of Brazil. Data collection took place electronically, through an instrument constructed using the SurveyMonkey program, containing a title, definition, and 19 risk factors for the nursing diagnosis. The data were analyzed using Fehring's method and descriptive statistics. The project was approved by a Research Ethics Committee.

**Results:**

title, definition and seven risk factors were validated as "very important": physical immobilization, pressure, surface friction, shearing forces, skin moisture,

alteration in sensation and malnutrition. Among the other risk factors, 11 were validated as "important": dehydration, obesity, anemia, decrease in serum albumin level, prematurity, aging, smoking, edema, impaired circulation, and decrease in oxygenation and in tissue perfusion. The risk factor of hyperthermia was discarded.

**Conclusion:**

the content validation of these components of the nursing diagnosis corroborated the importance of the same, being able to facilitate the nurse's clinical reasoning and guiding clinical practice in the preventive care for pressure ulcers.

## Introduction

Pressure Ulcers (PU) are lesions in the skin and/or underlying tissue, usually over a bony prominence, as a result of pressure or pressure in combination with shear and/or friction^1^.

International studies indicate rates of prevalence of pressure ulcers in American hospitals at around 12.3% among inpatients in clinical care units and 22% in intensive care units^2^. In Sweden, one General Hospital had a prevalence rate of PU of 23%^3^ while in Switzerland, the prevalence of PU of 26.5% was identified among hospitalized children^4^.

These data evidence that PU remain a frequent health problem with far-reaching effects, as they increase the risk of developing other health issues such as infections, osteomyelitis, septic arthritis and sepsis. PU cause patients great physical and emotional suffering, reducing their independence in daily activities and compromising their process of rehabilitation, and, consequently, having a negative impact on their quality of life^5^.

In addition to this, it is known that the financial costs are high for the health systems, associated with the acquiring of material for treating PU and their complications; these lead to more prolonged hospitalizations, with a need for more time spent on the care provided to patients with PU. These costs vary from US$2,000 to US$70,000 per wound, considering an annual average for the hospital varying from US$400,000 to US$700,000^5^.

In the light of this, preventing PU is shown to be essential, as it can impact positively on reducing the prevalence and incidence of this health issue and its complications and, therefore, reduces the costs of treating these. In this perspective and in the light of the absence of a Nursing Diagnosis (ND) clearly naming and defining the situation of risk for PU in the NANDA International (NANDA-I) Taxonomy II, Brazilian nurses undertook a study^6^ which contributed to the development of the ND of Risk for pressure ulcer. It was located in Domain 11- Safety/Protection, Class 2- Physical injury, recently published in the 2015 - 2017 edition of this diagnostic classification system^7^.

The content validation of the new ND was undertaken from when it was first constructed^6^, in order to evidence the reliability and degree of agreement in relation to the components which structure it; that is, title, definition and risk factors^8^ ^-^ ^10^. The studies on nursing diagnosis content validation are essential sources in searching for evidence and in the reduction of the probability of errors in the nurse's diagnostic process and decision-making process. As a result, in the present study, the objective was to validate the content of the components of the Nursing Diagnosis Risk for pressure ulcer (title, definition and risk factors), in accordance with specialists' opinion. 

## Method

This is a Diagnostic Content Validation (DCV) study of the components of the ND of Risk for pressure ulcer, through the opinion of specialists^11^. The sample was made up of 24 nurses, members of skin and wound care study groups, from five hospitals in the southern region of Brazil, and one in the southeastern region. The specialists were selected according to the following inclusion criteria: to participate or have participated in a skin and wound care study group for, at least, one year; to have had clinical practice in skin care, particularly in care for patients at risk for PU, for at least one year; to use a PU prevention and treatment protocol with application of the Braden scale as the instrument for predicting risk; and to respond to the instrument within the time period established of 60 days. Those nurses who met the inclusion criteria but who were absent from work during the period of the study due to holiday, absence and/or leave were excluded from the study. 

For data collection, the SurveyMonkey program was used, available free of charge on the Internet, in which was created a questionnaire with a link generated automatically, which was sent by email to the study participants. The responses were stored on the program's database. The first part of the instrument contained data on the specialists' characterization and professional and academic profile. The second part of the instrument focused on data of the DCV of the ND Risk for pressure ulcer, and contained the title and definition of this new ND, in which the specialists were to place an "X" on a five point Likert-type scale, covering one of the following possibilities: 1 - strongly disagree; 2 - disagree; 3 - do not know; 4 - agree, and 5 - strongly agree. Following that, the instrument presented the risk factors which made up the ND with their respective conceptual definitions and, in addition, a five point Likert scale in which the specialists were to mark one of the following alternatives: 1- does not indicate risk for PU; 2 - indicates little risk for PU; 3 - indicates moderate risk for PU; 4 - indicates high risk for PU and 5 - indicates a very high risk for PU. Along with the data collection instrument, the respondents were also sent an informative pamphlet on how to fill out the instrument and return it to the researcher, and on the ethical aspects of the study. The return of the filled-out instrument was taken as acceptance to participate in the study. 

The analysis of the variables related to the sample's characterization was undertaken through descriptive statistics on the SurveyMonkey program and using the IBM Statistical Package for the Social Sciences (SPSS) program, version 18.0. 

The analysis related to the ND's DCV was also statistical, taking into account the score attributed by the specialists to each one of its components and, based on that, the weighted average of the same indicated on the Likert scale with variation between 1 and 5 points, where: 1=0; 2=0.25; 3=0.50; 4=0.75 and 5=1^11^.

Any component (title, definition, risk factor) which received a mean greater or equal to 0.80 was considered "very important"; those with a mean below 0.80, but above 0.50, as "important"; and those with a mean equal to or less than 0.50 were discarded, as they were not considered important for this ND in the specialists' opinion^11^. The project was approved by the Research Ethics Committee, under Protocol 13-0034.

## Results

The study involved the participation of 24 specialist nurses, the large majority of whom were female (95.8%), with work in the area of nursing for a median time of 63.5 (20.75 - 183) months and with a median time of participation in study groups on skin and wounds of 48 (16.5 - 72) months. The academic title of the majority was specialist (58.3%), with them currently working in clinical care (54.2%). It was ascertained that they participated in events on the issue of prevention and treatment of PU, besides publication in annals and scientific articles (Table 1). 

**Table 1:** Characterization of the sample of specialist nurses (n=24). Porto Alegre, RS, Brazil, 2014.

Variable (n=24)			N (%)
Title			
	Ph.D		2 (8.3)
	M.A		4 (16.7)
	Specialist		14 (58.3)
	Graduate degree		4 (16.7)
Area of work			
	Clinical care		13 (54.2)
	Teaching of nursing		1 (4.2)
	Nursing management		5 (20.8)
	Other		5 (20.8)
Length of work in the area of nursing (in months)*			63,5 (20.75-183)
Length of participation in skin groups (in months)*			48 (16.5-72)
Participation in courses on pressure ulcers/skin lesions			
	Up to 10 hours		1 (4.2)
	From 10 to 20 hours		3 (12.5)
	From 20 to 30 hours		1 (4.2)
	From 30 to 40 hours		1 (4.2)
	Over 40 hours		18 (75)
Presentation of works in seminars/congresses/courses on pressure ulcers			
	Up to 10 hours		5 (20.8)
	From 10 to 20 hours		7 (29.2)
	From 20 to 30 hours		1 (4.2)
	From 30 to 40 hours		2 (8.3)
	Over 40 hours		2 (8.3)
Publications			
	Annals of congresses		
		1	3 (12.5)
		2	2 (8.3)
		More than 3	3 (12.5)
	Articles		
		1	1 (4.2)
		2	1 (4.2)
		3	1 (4.2)
		More than 3	2 (8.3)
	Chapters and/or books		
		1	5 (20.8)
		2	1 (4.2)

Source: Santos^7^.

*median (25%-75%)

The content validation of the ND Risk for pressure ulcer included the analysis of the title, definition and 19 risk factors which make up the same. 

The title and the definition were validated with a mean of ≥ 0.80 (Table 2).

**Table 2:** Title and definition validated by specialists for the ND Risk for pressure ulcer. Porto Alegre, RS, Brazil, 2014.

Components of the ND Risk for pressure ulcer	Mean
Title - Risk for pressure ulcer	0.92
Definition - Risk of tissue damage in the skin and underlying tissue, as a result of compression of the soft tissues generally over a bony prominence, during a time period capable of causing local ischemia and, consequently, necrosis	0.87

Source: Santos^7^.

Nineteen risk factors for the ND Risk for pressure ulcer were submitted to DCV. Seven (56.8%) were validated as "very important", with a mean of ≥ 0.80 (Table 3).

**Table 3:** Risk factors validated as "very important" for the ND Risk for pressure ulcer. Porto Alegre, RS, Brazil, 2014.

Risk factors for pressure ulcer validated as "very important"	Mean
Physical immobilization	0.97
Pressure	0.90
Shearing forces	0.90
Surface friction	0.89
Skin moisture	0.88
Malnutrition	0.84
Alteration in sensation	0.82

Source: Santos^7^.

Eleven (57.8%) risk factors were validated as "important", with a mean of > 0.5 and < 0.8 (Table 4).

**Table 4:** Risk factors validated as "important" for the ND Risk for pressure ulcer. Porto Alegre/RS, 2014.

Risk factors for pressure ulcer validated as "important"	Mean
Impaired circulation	0.78
Decrease in tissue perfusion	0.78
Dehydration	0.77
Decrease in tissue oxygenation	0.74
Edema	0.72
Obesity	0.70
Anemia	0.70
Prematurity	0.69
Decrease in serum albumin level	0.68
Aging	0.67
Smoking	0.54

Source: Santos^7^.

Only the risk factor of hyperthermia was discarded, as it received a mean of ≤ 0.50.

## Discussion

The DCV^11^ has been recognized by NANDA-I^7^ as an important method for refining the ND, with a level of evidence of 2.3, as it requires nurses' specialized opinion regarding the components of a nursing diagnosis. These studies seek to ascertain the reliability of the ND in practice, as well as considering its validity in relation to the degree of agreement regarding the components which structure it: title, definition, defining characteristics (signs and symptoms), related factors (etiology/cause) and risk factors. This type of study has been used both for developing new NDs and for refining those already existing, with a view to greater accuracy^7^.

Among the limitations of validation studies is the initial difficulty for defining the inclusion criteria for specialists, as there is no consensus in the literature in relation to the ideal number for the sample, besides the difficulty of finding nurse specialists in the areas of interest for investigation^8^ ^-^ ^10^. However, in the present study, the choice of the specialists was based on their academic background and, in particular, on the clinical experiences of the nurses who make up the different hospitals' study groups on care of the skin and tissues, so as to favor an accurate judgment on the components of the ND Risk for pressure ulcer. In addition to this, the diversity in the origin of the specialists extended the reliability of the data evaluated from different perspectives, showing there to exist, among the specialists, convergent opinions related to the physiopathology and risk factors for PU. 

The data referent to the validation of the title of the diagnosis "Risk for pressure ulcer" and of its definition "risk of cell damage in the skin and underlying tissue as a result of the compression of soft tissues, generally over a bony prominence, during a period of time capable of causing local ischemia and, consequently, necrosis" obtained means of > 80 points, that is to say, they were considered on the Likert scale as "strongly agree". 

This score referent to the title of the ND demonstrated agreement among the specialists, in addition to the same covering in a clear way the essential axes of an ND in accordance with NANDA-I^7^: 1 - focus of the diagnosis (in this case PU); 2 - subject of the diagnosis (when not made clear, this automatically comes to be the individual); 3 - judgment (combined in the diagnostic concept; in this case PU) and 7 - situation of the diagnosis (covered by the risk category). Similarly, the definition of the ND also presented a mean which showed agreement among the specialists, which demonstrates clarity and objectivity, based in the physiopathology and etiology of the PU.

The importance of a specific ND, with a clear title and definition regarding the risk of PU, has been evidenced by studies^12^ ^-^ ^14^ which have demonstrated that this clinical situation is common, both in patients at home and in hospitals, which justified the ND's development and inclusion in a diagnostic terminology, which supports the nurse in her management of the process of preventive care for this health issue. 

Seven risk factors (37%) were validated by the specialists as "very important", with a mean of >80 points: physical immobilization, pressure, shearing forces, surface friction, skin moisture, malnutrition and alteration in sensations.

The risk factor of physical immobilization was validated with a mean of 0.97, this being the highest score and agreement among the specialists, demonstrating this to be one of the principal factors increasing the patient's vulnerability to PU. It is known that reduced mobility increases the probability of greater time of pressure on the skin, favoring tissue ischemia and the occurrence of surface friction and shearing forces, with consequent possibility of breaking the skin and initiating ulceration^15^. Corroborating this idea, one transversal and exploratory study, with 43 older adults at risk of PU, hospitalized in clinical units of a Brazilian hospital, indicated - on the "activity" subscale of the Braden scale - that approximately 39.5% of these patients were bedridden or confined to a chair; on the subscale of "mobility", 60% of the patients were totally immobile or significantly limited, which explains these older adults' risk of developing PU^16^.

The risk factors related to pressure and shearing forces were validated with a mean of 0.90, and surface friction with a mean of 0.89. These external forces do not act in isolation, and cause the reduction of supply of blood to the skin and tissues. When associated with the patient's intrinsic factors (such as immobility, malnutrition and low tissue perfusion and oxygenation), they cause breaking of the skin due to ischemia, gradually increasing the development of the PU unless a prevention intervention is made. The repositioning of the patient, the use of polyurethane mattresses or air mattresses, the use of protective dressings on bony prominences and the constant assessment of humidity are examples of preventive interventions for PU^17^.

The risk factor of skin moisture received a mean of 0.88, reaffirming its importance for the development of PU. The exposure of the skin to humidity, principally to urine and feces, associated with abrasive forces such as surface friction and shearing forces, predisposes to an increase in irritation, causing maceration and ulceration and - once the PU is installed - the prognosis is negative regarding healing^14^.

The risk factor of malnutrition was validated with a mean of 0.84. Under conditions of weight loss, the musculature becomes hypertrophic, and the thin panniculus causes a break in the skin. With deficiency in nutrients, change also takes place in tissue healing, in the inflammatory reaction, and in the immune function when exposed to pressure. Poor nutrition can also be associated with low weight, indicated by the Body Mass Index (BMI <20), which favors the development of PU over the bony prominence, associated with pressure^13^ ^,^ ^18^ ^-^ ^19^.

The risk factor of alteration in sensations was validated with a mean of 0.82. Reduction in sensation occurs due to illnesses which trigger this form of harm, such as neurological ones, or through the use of analgesics and sedatives which, besides reducing sensation to physical stimulus, harm mobility. This is due to reduction of the normal stimulus to pain, leading the patient not to relieve prolonged pressure^19^.

Eleven risk factors were considered "important" by the specialists, with means between 50 and 80 points: impaired circulation, decrease in tissue perfusion, dehydration, decrease in tissue oxygenation, edema, obesity, anemia, prematurity, decrease in serum albumin level, aging and smoking. 

The risk factor of impaired circulation was validated with a mean of 0.78. Impairment in the peripheral circulation leads to reduction in local capillary pressure, with a negative impact on the nutrition of the tissues due to the deficient peripheral blood supply, with a probability of hypoxia, anoxia, and tissue ischemia. Peripheral vasoconstriction can be related to peripheral cardiovascular diseases, kidney diseases, anemia, arterial hypertension, diabetes mellitus, kidney and respiratory failure, concomitant infections, orthopedic lesions and use of medications, among other factors^20^. In the light of the numerous illnesses related to the circulation, this risk factor deserves attention and has been described in research on PU^(12, 21)^, principally among older adults, whose circulatory system is impaired by the characteristics of senescence.

The risk factor of dehydration was validated with a mean of 0.77. Dehydration impairs the vital functions of circulation, reducing the oxygenation of the tissues. It is known, furthermore, that deficit in ingesting liquids causes reduction in the skin turgor, this becoming increasingly fragile, which, coupled with the forces of abrasion (friction, pressure and shearing), increases the risk of ulceration^18^.

The risk factor of decrease in tissue oxygenation was validated with a mean of 0.74, and decrease in tissue perfusion with 0.78. Decrease in tissue perfusion and oxygenation reduces the rate of metabolism and energy of the tissue, predisposing to hypoxemia and organic dysfunction. Studies indicate that, in this situation, the patient is more predisposed to PU because of the deficit in perfusion and oxygenation, which can occur in situations such as trauma, loss of blood, and infection^22^.

The risk factor of edema was validated with a mean of 0.72. Edema is abnormal accumulation of liquid, in which there is increase in vascular permeability, and reduction in lymphatic drainage and, due to this, the tissue's circulation is compromised and it becomes poor in nutrients. When the tissue fluid increases and leaks outside the cells, the pressure on the blood vessels increases and, therefore, the blood flow and oxygenation of the tissues reduce, favoring ulceration^19^.

The risk factor of obesity was validated with a mean of 0.70. In obesity, there is the formation of adipose tissue, which reduces the vascularization of the skin surface, which can favor ischemia in the tissues and the development of PU, when some area of the body is subjected to pressure. Associated with this, the obese individual may have other comorbidities such as diabetes mellitus, making her still more vulnerable to PU^13^ ^,^ ^18^ ^-^ ^19^.

The risk factor of decrease in serum albumin level was validated with a mean of 0.68. Albumin is the most abundant protein in the plasma, used for determination of nutritional status. In low concentration, it causes changes in oncotic pressure and the formation of edema, which compromises the diffusion of oxygen and nutrients to the tissues, predisposing to hypoxia and cell death^13^ ^,^ ^18^.

The risk factor of anemia was validated with a mean of 0.70. This consists of the reduction in the quantity of hemoglobin in the blood stream, which is responsible for transporting oxygen to cells and tissues. The reduction of oxygen for the fibroblasts, cells responsible for healing of the tissues, reduces the formation of collagen and increases the tissue's susceptibility by precipitating ischemia and necrosis^13^ ^,^ ^19^. Supporting this, one study which described the profile of patients with PU, in a public hospital in São Paulo, indicated - among other factors - that the result of the laboratory tests presented a mean albumin of 2.7, glycemia of 169.7, hemoglobin of 9.5, leukocytes of 14.888 and C-reactive Protein of 79.2^22^. These data, related to the levels of serum albumin and hemoglobin, demonstrated the influence of the same in increasing the risk of PU. 

The risk factor of prematurity was validated with a mean of 0.69. It is known that the skin of a premature child (age between the 20^th^ and 37^th^ week of gestation) is fragile and that the physiological systems are not completely formed. There are deficiencies in oxygenation and vascularization of the skin and tissues, as well as in the integrity of the skin, with any break or ulceration being able to lead to systemic infection and increase in morbidity. In addition to this, hospitalized newborns in the critical units often require mechanical ventilation, cardiological monitoring and nutritional support, which hinders changing their position, favoring the increase of pressure and shearing on more vulnerable areas and, consequently, the development of PU^23^.

The risk factor of aging was validated with a mean of 0.67. It is known that the elderly population is considered at risk, due to its presenting decline in biological, psychic and social functions, as well as developing chronic degenerative diseases which cause prolonged periods of hospitalization and, later, of rehabilitation. As age advances, the skin becomes drier; as a consequence of the reduction in sweat and sebaceous glands, there is a reduction in vascularization and in properties such as perception of pain and the inflammatory response, besides there being hemodynamic changes and muscular atrophy, which causes the bony structures to become more prominent^24^. When these factors are associated with the morbid conditions and with other risk factors (such as changes in mobility, nutrition, and anal and urinary incontinence) the predisposition to developing PU increases. 

The risk factor of smoking was validated with a mean of 0.54. The nicotine present in cigarettes causes vasoconstriction, and, because of this, impedes the blood flow from occurring normally, hindering oxygenation and tissue perfusion, which favors necrosis and ulceration. In one case control study, undertaken in the United Kingdom, the cutaneous reactive response was assessed, after the installation of pressures in the sacral region, so as to identify the differences in the reactivity of blood flow in a group of individuals who were smokers and non-smokers, demonstrating that the smokers had a greater probability of forming tissue ischemia in comparison with non-smokers^25^. This datum strengthens the fact that PU is strongly related to the vascular risk factors brought by tobacco, corroborating what has been validated by the specialists in the present study. 

The risk factor of hyperthermia, although not validated by the specialists as important, as it presented a mean of < 0.50, is found described in the literature as a factor which favors the compromising of the body's metabolism, the instability of enzymatic functions, and the alteration of the metabolic pathways dependent on oxygen, causing reduction in the oxygenation of the tissues. This, associated with other concomitant factors such as immobility, malnutrition or obesity and extremes of age (prematurity or aging), makes the risk of PU imminent^18^.

The results obtained in this study were sent to the Diagnosis Development Committee (DDC) of the NANDA-I, responsible for analyzing proposals for new diagnoses for this taxonomy, and were approved and published in its most recent edition^6^ with some modifications such as maintaining the risk factor of hyperthermia.

## Conclusion

The DCV of the new Nursing Diagnosis Risk for Pressure Ulcer, undertaken by specialist nurses, demonstrated that its title, its definition and 18 of the 19 risk factors raised were considered to be important components of this ND. 

It is known that PU begin silently and are like icebergs; very dangerous below the surface, but unobtrusive on the surface. Thus, a specific and accurate ND for this clinical situation, with a clear definition and well-defined risk factors for this health issue, will assist the nurse in the process of clinical judgment, as well as supporting her in selecting preventive interventions which allow a favorable result, that is, the non development of the lesion. 

The short time for elaborating and submitting this ND to the NANDA-I DDC is considered to be a limiting factor for the study, bearing in mind that this taxonomy is updated every two years. Nevertheless, this ND's importance for the teaching of nursing is emphasized, given that its elements could contribute to the construction of logical reasoning regarding this clinical situation, as well as leading to further research such as the application of the same in real care environments, with results for qualifying the care. 

It is also understood that the classification systems with standardized language, such as NANDA-I, are instruments which favor the qualification of the nursing process, assist in clinical reasoning, and enable the better practice of nursing in the ambit of direct patient care, and in the communication, recording and managing of the care; and that, for this, it is necessary to refine and develop new elements such as the ND validated in this study. 
